# A Novel Function of Ethylene

**DOI:** 10.4137/grsb.s2202

**Published:** 2009-04-07

**Authors:** Aiko Amagai

**Affiliations:** Department of Biomolecular Science, Graduate School of Life Sciences, Tohoku University, Katahira 2-1-1, Aoba-Ku, Sendai 980-8577, Japan

**Keywords:** ethylene, cAMP, zyg1, zygote, macrocyst, Dictyostelium

## Abstract

The cellular slime mold, *Dictyostelium mucoroides-7* (Dm7) exhibits clear dimorphism; macrocyst formation as a sexual process and sorocap formation as an asexual process. These two life cycles are regulated by two regulators, ethylene and cyclic AMP (cAMP). This is the first report demonstrating a novel function of ethylene at the cellular level. That is, ethylene induces a zygote formed by cell fusion and subsequent nuclear fusion. Recently, the function of ethylene at the molecular level has been clarified as it induces zygote formation through an enhanced expression of a novel gene, *zyg1*. The signaling pathway for induction or inhibition of zygote formation is now trying to be clarified focusing on the ZYG1 protein.

## Background

The cellular slime mold is a microorganism living in the soil. It has some interesting features. The amoeboid cells grow and multiply feeding on bacteria as a food supply at the vegetative growth phase. Upon exhaustion of the bacterial food supply (starvation), however, starving cells gather together by chemotactic movement toward cAMP, forming aggregation streams. A tip is formed on the top of each cell aggregate, which then migrates as a slug-shaped mass. After the migration, the slug changes dramatically its shape to form a sorocarp consisting of a stalk with an apical mass of spores. Thus, the differentiation phase of the cellular slime mold is separated from the growth phase. Furthermore, they have only two cell types of differentiation, that is, stalk and spore cells. These characters are quite suitable for studies of differentiation including the mechanisms of the transition from growth to the differentiation phase. As they are usually haploid, it is easier to manipulate genes, such as clone, knockout genes and so on. For this reason, the cellular slime mold is regarded as a model organism.

**Figure 1:**
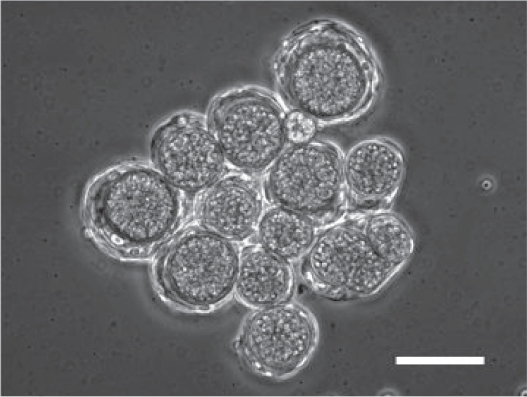
A cluster of macrocysts formed under submerged conditions Dm7 cells form macrocysts under submerged conditions or in the dark. Each macrocyst is surrounded by a thick wall. Granules observed inside macrocysts are endocytes that are cells engulfed by zygotes. Bar:100 um.

Some species in cellular slime molds have another life cycle, called macrocyst formation. Macrocyst formation is known as a sexual process, while sorocarp formation as an asexual process. *Dictyostelium mucoroides 7* (Dm7), one of the species of cellular slime molds, forms macrocysts homothallically. Macrocyst formation in Dm7 is characterized by the formation of large aggregates after starvation, which are subdivided into smaller masses (precysts), each of which is surrounded by a fibrillar sheath. At the center of each precyst there arises a cytophagic cell (giant cell), which in turn engulfs all the other cells in the precyst. The engulfed cells (endocytes) are eventually broken down into granular remnants. The enlarged cytophagic cell finally becomes surrounded by a thick wall to form the mature macrocyst (Fig. 1).^1^ After a resting period, the macrocyst germinates to release several amoeboid cells and initiates a new life cycle.^2^ During the study to clarify the mechanism by which the developmental fate is decided, sorocarp or macrocyst formation, we have identified ethylene as one of the regulators.^3^

Ethylene is well known as a potent plant hormone, which controls almost all aspects of development in plants, including sex expression, fruit ripening, senescence and responses to wounding.^4^,^5^ Since the receptor of ethylene has been reported, the signaling pathways involving ethylene have been actively studied.^6^,^7^,^8^ In this review, we have described a novel function of ethylene that has been clarified in a sexual cell cycle of *Dictyostelium* cells, macrocyst formation.

## Macrocyst Formation is a Sexual Process in Cellular Slime Molds

Macrocyst formation was first reported by Brefeld in 1869.^9^ Its process was regarded as the sexual process in cellular slime molds. In fact, there are three kinds of mating types in the macrocyst formation; heterothallic, homothallic and bi-sexual mating types.^10^,^11^ In *Dictyostelium discoideum* (Dd), the wild-type NC4 cells undergo to mate with an opposite mating type, V12M2 cells heterothallically. Dm7, one of the strains of *Dictyostelium mucoroides* (Dm) has not a mating type and mates by itself homothallically. Since Raper^12^ described the process and sexuality of macrocyst formation, a lot of evidence concerning the sexuality of macrocysts has been accumulated.^13^–^15^ The sexual process is recognized by the appearance of a zygote which is formed by the cell fusion and subsequent nuclear fusion. If cellular events such as cell fusion, nuclear fusion and meiosis are observed in the process of macrocyst formation, it is certainly recognized as the sexual process in cellular slime molds. Although the period of meiosis still remains to be specified, the synaptonemal complex which is formed at the late leptotene stage during meiosis appears during macrocyst formation in some strains of cellular slime molds, such as *Polysphondylium violaceum* (Pv)^16^ and Dd.^13^ Furthermore, from various hybridization breedings, the occurrence of recombinants during macrocyst formation has been realized in Dm7, its mutant,^17^ the heterothallic strains of *D. giganteum* (Dg),^18^ *P. pallidum* (Pp) and Dd.^19^,^20^ These results suggest that meiosis may occur during macrocyst formation.

Recently, giant cells formed during macrocyst formation in Dm 7 have been proved to be zygotes that are produced by cell fusion and subsequent nuclear fusion.^15^ When Dm 7 cells were allowed to develop in the dark and stained with diamino-2-phenylindole (DAPI), giant cells containing two nuclei were observed. The giant cells containing an enlarged and brightly stained nucleus were also noticed. Neither binucleate cells nor giant cells were formed under the conditions favouring sorocarp formation. In order to know whether the binucleate cell is a true zygote produced by the fusion of two cells, cells vitally stained with fluorescein isothiocyanate (FITC) were mixed with cells vitally stained with DAPI at a 1:1 ratio and developed until the time when giant cells appeared. Although most of the cells were stained with either FITC or DAPI, a small number of cells was larger in size and stained with both of the fluorescent dyes. This clearly indicates that these giant and double-stained cells are formed by cell fusion. As the binucleate cells and the double-stained cells were observed at almost the same developmental stage, the observed binucleate cells are presumably zygotes formed by cell fusion. An enlarged and brightly stained nucleus in the giant cell contained twice the amount of DNA, resulting from nuclear fusion. The evidence of cell fusion and nuclear fusion occurring during macrocyst formation has been also reported in heterothallic strains. The moment when two gametes fused has been photographed by a time lapse video recorder.^21^ The appearance of nuclei containing a two-fold DNA content has been also noticed.^13^ These results show that the giant cell is a true zygote produced by cell fusion and subsequent nuclear fusion, beyond the difference of mating types, homothallically or heterothallically.

## The Function of Ethylene in Macrocyst Formation at a Tissue Level

The two life cycles, the sorocarp and the macrocyst formation, are regulated by several environmental conditions, such as light and water. Dm7 cells form sorocarps in the light, whereas they form macrocysts in the dark or in the water. These environmental conditions affect the synthesis of chemical regulators within the cells. The two chemicals, ethylene and cAMP, have been demonstrated as chemical regulators for choice of developmental pathways in Dm7.^3^,^22^ It had been already reported that volatile substance(s) might be involved in the macrocyst formation.^23^ Filosa and his colleague proposed two gases, carbon dioxide (CO_2_) and charcoal-absorbed gases (CAG), as candidates of chemical regulators in Dm7. When the gases were absorbed by charcoal, only sorocarps were formed instead of macrocysts.^23^,^24^ On the other hand, as macrocysts were formed by absorbing CO_2_ gas with KOH, CO_2_ could be an antagonist of CAG. Underwater favors macrocyst formation in contrast to sorocarp formation which predominantly takes place in mineral oil. This suggested that CAG might have some hydrophobic characters. Based on the idea that a hydrophobic gas (es) produced by cells may accumulate around cells at higher levels due to its slow diffusion into water and act antagonistically to CO_2_, we speculated on ethylene as a potent candidate of CAG. The possibility that ethylene might actually act as CAG was tested using MF1 cells, a spontaneous mutant isolated from Dm7. The mutant MF1 cells are able to form macrocysts even in the light depending upon cell densities plated. This suggested that CAG might have the threshold concentration to induce macrocyst formation. In fact, MF1 cells failed to form macrocysts even at higher cell densities, when they were allowed to develop in a larger incubation chamber. It was supposed that the concentration of CAG produced by the cells would be insufficient for induction of macrocysts by being diluted in the larger chamber. However, MF1 cells changed their developmental forms from sorocarps to macrocysts even in the larger chamber by the addition of ethylene. As was expected, inhibitors of ethylene biosynthesis, such as aminooxyacetic acid (AOA) and aminoethoxyvinyl glycine (AVG) greatly inhibited macrocyst formation. Ethylene production by MF1 cells and Dm7 cells was confirmed by gas chromatography.^3^ From these results, ethylene was finally identified as a CAG, an inducer of macrocyst formation. When Dm7 cells formed sorocarps in the light, it was found that a significant amount of ethylene was produced from the Dm7 cells. Why did Dm7 cells not form macrocysts in the light, though they produced ethylene? Interestingly, when MF1 and Dm7 cells were mixed at the ratio of 1:1 and developed in the light, MF1 as well as Dm7 formed sorocarps though their cell densities were sufficient to form macrocysts by themselves. These facts raised the possibility that Dm7 cells might produce the second regulator(s) which competes with the ethylene action and shifts the developmental fate from macrocyst to sorocarp formation. Cyclic AMP was identified as a second regulator, because the developmental fate was changed from macrocyst to sorocarp formation in the presence of cAMP.^22^ Therefore, the choice of developmental pathways seemed to be determined by the balance of cAMP and ethylene amounts at the aggregate stage when the developmental fate was determined. This was confirmed by determination of the amount of two regulators produced at the aggregation stage.^25^ The amount of cAMP was higher in the sorocarp than in the macrocyst formation, whereas, the amount of ethylene production was decreased when sorocarps were formed in the presence of AOA. The tight relationship between the amount of ethylene and the induction of macrocyst formation was directly confirmed, using two kinds of transformants over- and under-producing ethylene.^26^ *Dd-aco*, an 1-aminocyclopropane-1-carboxylic acid (ACC) oxidase homologue gene, isolated from Dd (DDBJ, EMBL and Gen-Bank databases with the accession no. AB105858) was introduced into Dm7 cells to produce transformants. Transformant over-producing *Dd-aco* (ACO^OE^) actually produced a larger amount of ethylene than the wild type Dm7, whereas transformant under-producing *Dd-aco* (ACO-RNAi) produced a smaller amount of ethylene than Dm7. ACO^OE^ cells formed macrocysts, while ACO-RNAi cells failed to form macrocysts independently of culture conditions. The volatile substance(s) released from NC4 cells was also shown to induce macrocyst formation in V12M2 without its mating type NC4 cells.^27^ Since the production of ethylene by NC4 was confirmed by gas chromatography,^28^,^29^ it is quite likely that the volatile substance produced by NC4 cells is ethylene and induces the macrocyst formation in V12M2 cells, as in the case of Dm7.

Concerning the biosynthesis of ethylene in *Dictyostelium*, it has been supposed that ethylene is synthesized from methionine through S-adenosyl-L-methionine (SAM) and ACC as in the case of higher plants.^29^ The presence of an ACC oxidase homologue gene and ACC synthase homologue gene (DDBJ, EMBL and GenBank databases with the accession no. GO274713) in *Dictyostelium* also suggested the pathway of ethylene biosynthesis in *Dictyostelium* as the same as in higher plants. Recently, the existence of ethylene receptor in *Dictyostelium* was suggested by the use of 1-methylcyclopropene (1-MCP), a specific inhibitor of ethylene action through its specific binding to the ethylene receptor, though the ethylene receptor in *Dictyostelium* has not been identified yet.^26^ Thus, *Dictyostelium* cells must share many cellular aspects with higher plants through the ethylene receptor.

## The Function of Ethylene in Zygote Formation at a Cellular Level

**Figure 2:**
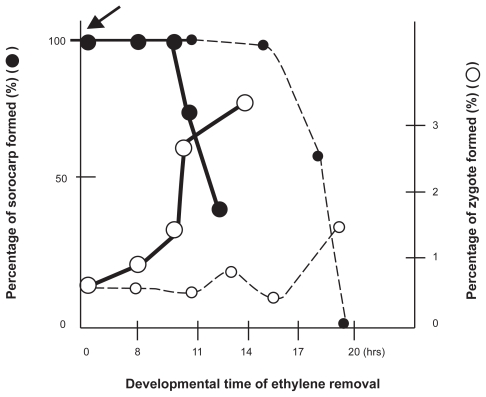
Developmental kinetics showing sorocarp and binucleate cell formation Ethylene was removed by charcoal at designated developmental times. Percentages of sorocarps formed (●) were determined after 48 hrs of incubation. 100% of sorocarps indicate only sorocarps were formed on the agar plates, while 0% of sorocarps indicate only macrocysts were formed on the agar plates. The percentages of zygotes (○) indicate the number of binucleate cells stained with DAPI as an index of zygotes. Dotted lines show the kinetics obtained without the application of ethylene, while solid lines show the kinetics obtained with the application of ethylene at the beginning of incubation. The time of ethylene application was indicated by an arrow. These results indicate that the time acquired non-necessity of ethylene for macrocyst formation advances in concert with the advanced and increasing formation of binucleate cells. modified data cited from.^15^

There are several cellular events occurring in the process of macrocyst formation, such as zygote formation and engulfment of the other cells by the zygote in a cell mass, etc. Which of the cellular events are controlled by ethylene? In order to determine the developmental stages when ethylene is effective to induce macrocyst formation, ethylene gas produced by Dm7 and MF1 cultures was removed by use of charcoal at various times of development. As a result, the developmental fate was shifted from macrocyst to sorocarp formation by removal of ethylene at the early developmental stages. This means that ethylene is necessary for macrocyst formation at the early developmental stages. However, ethylene becomes unnecessary for macrocyst formation at the later developmental stages. Since the percentage of sorcarp formation changes in inverse proportion to the ratio of binucleate cells, the timing when ethylene becomes unnecessary for macrocyst formation is consistent with the appearance of binucleate cells regarded as zygotes. The developmental time acquired non-necessity of ethylene for macrocyst formation was delayed when the cell densities plated were decreased. However, when ethylene was applied in the culture dishes at the beginning of this culture, the time acquired non-necessity of ethylene for macrocyst formation advanced in concert with the advanced and increasing formation of binucleate cells (Fig. 2).^15^ These results strongly suggest that ethylene may directly induce zygote formation. As was expected, cAMP, the second regulator, exhibits an inhibitory effect on the process of zygote formation.^30^ When the number of binucleate cells among cells stained with DAPI was counted as an index of zygote, the number of binucleate cells was decreased by the addition of cAMP, while it was increased by the addition of phosphodiesterase (PDE). In conclusion, two regulators, ethylene and cAMP, regulate directly zygote formation during macrocyst formation.

**Figure 3:**
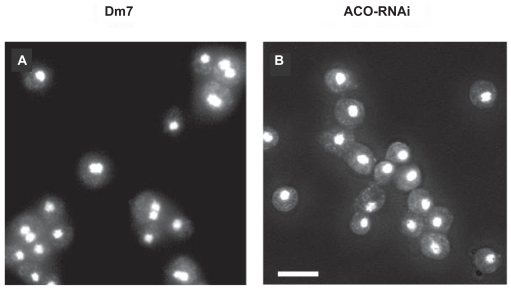
Zygote formation in Dm7 and ACO-RNAi cells Dm7 and ACO-RNAi cells were developed under submerged conditions for 10 hrs after starvation. They were stained with DAPI after fixation with methanol. Many binucleate cells are observed in Dm7 cells (**A**), while not in ACO-RNAi cells (**B**). Thus, it is evident that ACO-RNAi cells fail to form binucleate cells because of the decreased production of ethylene. Bar:10 um.

The close relation between the amount of ethylene produced and zygote formation was also shown clearly using the transformant: ACO-RNAi cells never formed zygotes as was expected (Fig. 3).^26^ In heterothallic strains, zygote formation is also regulated by ethylene and cAMP. It was induced by ethylene, while it was inhibited by cAMP.^31^,^32^ Accordingly, ethylene and cAMP may act on the regulation of zygote formation beyond the difference of mating types. Ethylene functions in the other cellular events occurring during macrocyst formation remain to be elucidated.

## The Function of Ethylene in the Cell Fusion at a Molecular Level

How does ethylene lead to cell fusion occurring during zygote formation? To address this question, we attempted to find out what molecules are related to signaling pathways involved in the cell fusion during zygote formation. Two strategies were adopted to find out the molecules. 1) Identification of chemicals involved in the signaling pathways for zygote formation. 2) Identification of genes which are specifically expressed in the process of zygote formation.

### Identification of chemicals involved in the signaling pathways for zygote formation

It is well known that calcium ion (Ca^2+^) plays an important role in cell fusion in many organisms.^33^,^34^ Ca^2+^ was also proposed to be a critical factor for zygote formation including cell fusion in *Dictyostelium*. The percentage of zygotes was elevated by the presence of extracellular Ca^2+^.^35^,^36^,^30^ Phorbol esters such as 12*O*-tetradecanoylphorbol-13-acetate (TPA), potent activators of protein kinase C (PKC), have been reported to enhance the formation of zygotes.^37^ In contrast, staurosporine, an inhibitor of kinases including PKC, inhibited zygote formation^37^ and macrocyst formation.^38^ Taken together these results suggested that the signaling pathway involving Ca^2+^ and PKC would act on the induction of zygote formation. It has been documented that the signaling pathway including Ca^2+^ and PKC is involved in cell fusion during myogenesis.^33^,^39^,^40^ Cell fusion as realized in fertilization and myogenesis might share the Ca^2+^ and PKC-mediated signaling pathway.

To examine involvements of other kinases in zygote formation, effects of some kinase inhibitors on macrocyst formation were tested. We have demonstrated that calmodulin and cAMP-dependent kinase (PKA) inhibit zygote formation through the signaling pathway triggered by cAMP.^38^ Lydan and O’Day^41^ have proposed evidence showing roles of calmodulin as both a negative (gamete formation) and a positive (cell fusion) regulator of sexual events in Dd. Calmodulin dependent phosphorylation and dephosphorylation has been also reported by Lydan and O’Day.^42^ They have summarized the roles of Ca^2+^ and calmodulin in signal transduction pathway in their book.^43^ However, proteins phosphorylated or dephosphorylated in a calmodulin-dependent manner as well as the target proteins of PKA remain to be identified.

### Identification of genes which are specifically expressed in the process of macrocyst formation

**Figure 4:**
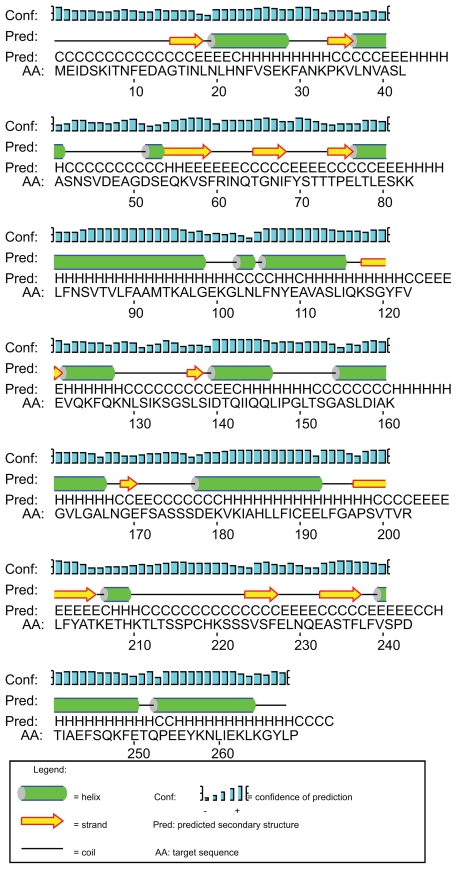
PSIPRED graphical output from prediction of ZYG1 produced by PSIPRED View-a Java visualization tool that produced two-dimensional graphical presentations of PSIPRED predictions.^47^,^48^

**Figure 5:**
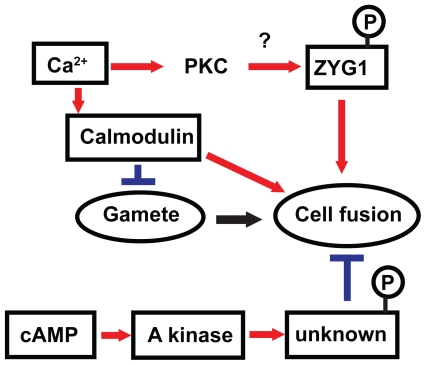
Diagram showing the signaling pathways involved in zygote formation By several pharmacological experiments, it has been suggested that protein kinase A (PKA) is involved in the downstream of cAMP as an inhibitory signaling pathway together with calmodulin activated by Ca^2+^. Lydan and O’Day^41^ have shown evidence about the role of calmodulin as both a negative (gamete formation) and a positive (cell fusion) regulator of sexual events in *Dictyostelium.* It is most likely that ZYG1 may be phosphorylated by PKC activated by Ca^2+^ in the signaling pathway inducing zygote formation. : induction → : inhibition ⫞

Using differential screening of genes, the *zyg1* gene was isolated from Dm7 cells as a novel gene expressed predominantly during macrocyst formation (DDBJ/EMBL/GenBank, accession no. AB006956).^44^ The predicted protein, ZYG1, consists of 268 amino acids with a molecular mass of 29.4 kDa. After BLAST^45^ and FASTA^46^ searches, the amino acid sequence as a whole shows no convincing similarity to the known proteins. Although the ZYG1 protein is predicted to have several sites phosphorylated by PKC, it has neither transmembrane domains nor specific signal sequences. The second structure of ZYG1 shown in Figure. 4 is predicted by PSIPREDView-a Java visualiztion tool.^47^,^48^ The expression of *zyg1* gene began 2 hrs after starvation, reached the maximum level at 8 hrs, and decreased when Dm7 cells were cultured under submerged conditions. This expression pattern is quite similar to the developmental kinetics of zygote formation with about 1 hr precedence. The number of zygote began to increase 5 hrs after starvation, reached the maximum level at 9 hrs and then gradually decreased.^38^ From these observations, the *zyg1* gene was predicted to be involved in zygote formation. In order to confirm this prediction, the *zyg1* gene was introduced into Dm7 cells. The transformant overexpressing the *zyg1* gene formed macrocysts on agar even in the light which is the condition favourable to sorocarp formation. In addition, they formed many giant cells besides macrocysts. These results suggested that the *zyg1* gene might be involved in the induction of zygote formation. The fact that ZYG1 protein has several predicted phorphorylation sites by PKC raised the possibility that ZYG1 protein itself could be a candidate of the substrate of PKC. In general, activated PKC is known to be translocated to the cell membrane. ZYG1 protein is supposed to be translocated from the cytosol to the cell membrane, provided that ZYG1 protein is phosphorylated by PKC. The signaling pathways involved in zygote formation are summarized in Figure. 5. Since some genes which might be involved in cell fusion have been isolated also from Dd,^49^ clarification of their precise functions is promising.

## The Relationship between Ethylene and ZYG1

**Figure 6:**
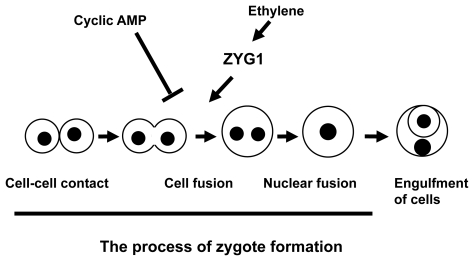
Diagram showing the induction and inhibition of zygote formation by ethylene, cAMP and ZYG1 During macrocyst formation, there are several cellular events such as cell-cell contact, cell fusion and engulfment of cells. Cell fusion occurring during zygote formation is induced by ethylene through an enhanced expression of *zyg1* gene, while it is inhibited by cAMP.

Ethylene and ZYG1 have a similar function in zygote formation. In order to know the relation between the two, the expression of *zyg1* gene during the development in ACO^OE^ cells overproducing ethylene and ACO-RNAi cells under-producing ethylene was examined and compared. The results obtained showed that Dm7 cells and ACO^OE^ cells exhibited higher levels of *zyg1* expression, while ACO-RNAi cells exhibited significantly lower levels of expression, coupling with their failure to form zygotes. The reason why no difference of *zyg1* expression between Dm7 and ACO^OE^ cells is detected might be due to developmental conditions. The submerged conditions used for extraction of mRNA are favorable to zygote formation. In fact, both Dm7 and ACO^OE^ cells formed macrocysts under submerged conditions. Importantly, *zyg1* expression was decreased by application of AOA, an inhibitor of ethylene biosynthesis, into the culture medium. Taken together these results indicate that ethylene induces zygote formation through an enhanced expression of *zyg1*^26^ (Fig. 6).

## Perspective

The functions of ethylene in *Dictyostelium* have been clarified at the multicellular, cellular and molecular levels as summarized in Table 1. The ZYG1 protein induced by ethylene will be focused on to know the signaling pathways involved in the sexual cell fusion occurring in zygote formation. We are now speculating that ZYG1 may be translocated into the cell membrane and then phosphorylated by PKC for the induction of cell fusion. These issues will become clear soon.

## Figures and Tables

**Table 1:** Summary of ethylene functions in *Dictyostelium*.

Levels of function	Events induced by ethylene	References
Tissue level	Macrocyst formation	Amagai^3^
Cellular level	Cell fusion occurring in zygote formation	Amagai^15^
Molecular level	Expression of *zyg1* gene	Amagai et al.^26^

Ethylene induces macrocyst formation by stimulating cell fusion occurring in zygote formation. The zygote formation is induced through an enhanced expression of a novel gene, *zyg1*.

## References

[1] Filosa MF Dengler RE Ultrastructure of macrocyst formation in the cellular slime mold Dictyostelium mucoroides: Extensive phagocytosis of amoebae by a specialized cell Dev Biol 1972 29 1 16 4342276 10.1016/0012-1606(72)90038-3

[2] Nickerson AW Raper KB Macrocysts in the life cycle of the Dictyosteliaceae. II. Germination of the macrocyst Amer J Bot 1973 60 247 53

[3] Amagai A Induction by ethylene of macrocyst formation in the cellular slime mould Dictyostelium mucoroides J Gen Microbiol 1984 130 2961 5

[4] Abels FB Ethylene in plant biology Academic Press 1973

[5] Abeles FB Morgan PW Saltveit MEJ Ethylene in plant biology2nd ed Academic Press 1992

[6] Bleecker AB Estelle MA Somerville H Insensitivity to ethylene conferred by a dominant mutation in Arabidopsis thaliana Science 1988 241 1086 9 17747490 10.1126/science.241.4869.1086

[7] Johnsons PR Ecker JR The ethylene gas signal transduction pathway: a molecular perspective Annu Rev Genet 1988 32 227 54 10.1146/annurev.genet.32.1.227 9928480

[8] Klee HJ Ethylene signal transduction. Moving beyond Arabidopsis Plant Physiol 2004 135 660 7 15208412 10.1104/pp.104.040998 PMC514102

[9] Brefeld O Dictyostelium mucoroides. Ein neuer Organismus aus der Verwandshaft der Myxomyceten Abhandl Senckenbergish Naturf Ges 1869 7 85 107

[10] Clark MA Francis D Eisenberg R Mating types in cellular slime molds BBRC 1973 52 672 8 4736370 10.1016/0006-291x(73)90765-1

[11] Erdos GW Raper KB Vogen LK Mating types and macrocyst formation in Dictyostelium discoideum Proc Natl Acad Sci U S A 1973 70 1828 30 16592095 10.1073/pnas.70.6.1828 PMC433606

[12] Raper KB The Dictyostelids Press Princeton 1984

[13] Okada H Hirota Y Moriyama R Nuclear fusion in multinucleated giant cells during the sexual development of Dictyostelium discoideum Dev Biol 1986 118 95 102

[14] O’Day DH Tama RA Lydan MA Gamete formation reflects the sexual pheromone hierarchy of Dictyostelium giganteum Experientia1987a 43 619 21

[15] Amagai A Induction of zygote formation by ethylene during the sexual development of the cellular slime mold Dictyostelium mucoroides Differentiation 1989 41 176 83

[16] Erdos GW Nickerson AW Raper KB Fine structure of macrocysts in Polyspondylium violaceum Cytobiologie 1972 6 351 66

[17] MacInnes MA Francis D Meiosis in Dictyostelium mucoroides Nature 1974 251 321 3 4473717 10.1038/251321a0

[18] Erdos GW Raper KB Vogen LK Sexuality in the cellular slime mold Dictyostelium giganteum Proc Natl Acad Sci U S A 1975 72 970 3 1055396 10.1073/pnas.72.3.970 PMC432445

[19] Francis D Macrocyst genetics in Polysphondylium pallidum, a cellular slime mould J Gen Microbiol 1975 89 310 8 1236929 10.1099/00221287-89-2-310

[20] Wallace MA Raper KB Genetic exchanges in the macrocysts of Dictyostelium discoideum J Gen Microbiol 1979 113 327 37 292755 10.1099/00221287-113-2-327

[21] O’Day DH McConachie DR Rivera J Appearance and developmental kinetics of a unique cell type in Dictyostelium discoideum: It is the gamete phase of sexual development J Exp Zool1987b 242 153 9

[22] Amagai A Filosa MF The possible involvement of cyclic AMP and volatile substance(s) in the development of a macrocyst-forming strain of Dictyostelium mucoroides Dev Growth Differ 1984 26 583 9 10.1111/j.1440-169X.1984.00583.x 37281137

[23] Filosa MF Macrocyst formation in the cellular slime mold Dictyostelium mucoroides: Involvement of light and volatile morphogenetic substance(s) J Exp Zool 1979 207 491 5

[24] Weinkauf AM Filosa MF Factors involved in the formation of macrocyst by the cellular slime mold, Dictyostelium mucoroides Can J Microbiol 1965 11 385 7 14323052 10.1139/m65-048

[25] Amagai A Regulation of the developmental modes in Dictyostelium mucoroides by cAMP and ethylene Differentiation 1987 36 111 5 2834253 10.1111/j.1432-0436.1987.tb00184.x

[26] Amagai A Soramoto S Saito S Ethylene induces zygote formation through an enhanced expression of *zyg1* in Dictyostelium mucoroides Exp Cell Res 2007 313 2493 503 17499244 10.1016/j.yexcr.2007.04.012

[27] Lewis KE O’Day DH Sexual hormone of Dictyostelium disocideum in volatile Nature 1977 268 730 1

[28] Bonner JT Humoral Control of Growth and Differentiation Academic Press 1973

[29] Amagai A Maeda Y The ethylene action in the development of cellular slime molds: an analogy to higher plants Protoplasma 1992 167 159 68

[30] Suzuki T Amagai A Maeda Y Cyclic AMP and Ca^2+^ as regulators of zygote formation in the cellular slime mold Dictyostelium mucoroides Differentiation 1992 49 127 32 1319932 10.1111/j.1432-0436.1992.tb00660.x

[31] O’Day DH Lyden MA The regulation of membrane fusion during sexual development in Dictyostelium discoideum Biochem Cell Biol 1989 67 321 6 (Review) 2675932 10.1139/o89-050

[32] Amagai A Induction of heterothallic and homothallic zygotes in Dictyostelium discoideum by ethylene Dev Growth Differ 1992 34 293 9 10.1111/j.1440-169X.1992.tb00018.x 37282260

[33] Shainberg A Yagil G Yaffe D Control of myogenesis in vitro by Ca^2+^ concentration in nutritional medium Exp Cell Res 1969 58 163 7 5404063 10.1016/0014-4827(69)90127-x

[34] Ishihara K Hosono J Kanatani H Toad egg-jelly as a source of divalent cations essential for fertilization Dev Biol 1984 105 435 42 6434358 10.1016/0012-1606(84)90300-2

[35] Chagla AH Lewis KE O’Day DH Ca^2+^ and cell fusion during sexual development in liquid cultures of Dictyostelium discoideum Exp Cell Res 1980 126 501 5 10.1016/0014-4827(80)90298-0 7363960

[36] Szabo SP O’Day DH Chagla AH Cell fusion, nuclear fusion, and zygote differentiation during sexual development of Dictyostelium discoideum Dev Biol 1982 90 375 82 6804289 10.1016/0012-1606(82)90387-6

[37] Gunther KE Ramkisson H Lydan MA Fertilization in Dictyostelium discoideum: pharmacological analysis and the presence of a substrate protein suggest protein kinase C is essential for gamete fusion Exp Cell Res 1995 220 325 31 7556440 10.1006/excr.1995.1322

[38] Kawai S Maeda Y Amagai A Promotion of zygote formation by protein kinase inhibitors during the sexual development of Dictyostelium mucoroides Dev Growth Differ 1993 35 601 7 10.1111/j.1440-169X.1993.00601.x 37282253

[39] Paterson B Strohman RC Myosin synthesis in cultures of differentiating chicken skeletal muscle Dev Biol 1972 29 113 38 4672726 10.1016/0012-1606(72)90050-4

[40] David JD Faser CR Perrot GP Role of protein kinase C in chick embryo skeletal myoblast fusion Dev Biol 1990 139 89 99 2328843 10.1016/0012-1606(90)90281-m

[41] Lydan MA O’Day DH Different developmental functions for calmodulin in Dictyostelium: Trifluoperazine and R24571 both inhibit cell and pronuclear fusion but enhance gamete formation Exp Cell Res 1988 178 51 63 3409979 10.1016/0014-4827(88)90377-1

[42] Lydan MA O’Day DH Calmodulin-dependent phosphorylation and dephosphorylation during fertilization in Dictyostelium discoideum BBRC1993a 192 1073 8 8389540 10.1006/bbrc.1993.1526

[43] Lydan MA O’Day DH O’Day DH Signal transduction and cell fusion in Dictyostelium: calcium, calmodulin, and an endogenous inhibitor Signal transduction during biomembrane fusion San Diego Academic Press 1993b 245 63

[44] Amagai A Involvement of a novel gene, *zyg1*, in zygote formation of Dityostelium mucoroides J Muscle Res and Cell Motility 2002 23 867 74 10.1023/a:1024448316675 12952084

[45] Altschul SF Gish W Miller W Basic local alignment search tool J Mol Biol 1990 215 403 10 2231712 10.1016/S0022-2836(05)80360-2

[46] Pearson WR Rapid and sensitive sequence comparison with FASTP and FASTA Method Enzymol 1990 183 63 98 10.1016/0076-6879(90)83007-v 2156132

[47] Jones DT Protein secondary structure prediction based on position-specific scoring matrices J Mol Biol 1999 292 195 202 10493868 10.1006/jmbi.1999.3091

[48] McGuffin LJ Bryson K Jones DT The PSIPRED protein structure prediction server Bioinformatics 2000 16 404 5 10869041 10.1093/bioinformatics/16.4.404

[49] Urushihara H Muramoto T Genes involed in Dictyostelium discoideum sexual reproduction Eur J Cell Biol 2006 85 961 8 16815590 10.1016/j.ejcb.2006.05.012

